# Novel Real-Time Diagnosis of the Freezing Process Using an Ultrasonic Transducer

**DOI:** 10.3390/s150510332

**Published:** 2015-05-04

**Authors:** Yen-Hsiang Tseng, Chin-Chi Cheng, Hong-Ping Cheng, Dasheng Lee

**Affiliations:** Department of Energy and Refrigerating Air-Conditioning Engineering, National Taipei University of Technology, Taipei 10608, Taiwan; E-Mails: yhtntut@gmail.com (Y.-H.T.); hpcheng7@gmail.com (H.-P.C.); f11167@ntut.edu.tw (D.L.)

**Keywords:** freezing point, freezing process diagnosis, ultrasonic transducer (UT), cooling/freezing periods

## Abstract

The freezing stage governs several critical parameters of the freeze drying process and the quality of the resulting lyophilized products. This paper presents an integrated ultrasonic transducer (UT) in a stainless steel bottle and its application to real-time diagnostics of the water freezing process. The sensor was directly deposited onto the stainless steel bottle using a sol-gel spray technique. It could operate at temperature range from −100 to 400 °C and uses an ultrasonic pulse-echo technique. The progression of the freezing process, including water-in, freezing point and final phase change of water, were all clearly observed using ultrasound. The ultrasonic signals could indicate the three stages of the freezing process and evaluate the cooling and freezing periods under various processing conditions. The temperature was also adopted for evaluating the cooling and freezing periods. These periods increased with water volume and decreased with shelf temperature (*i.e.*, speed of freezing). This study demonstrates the effectiveness of the ultrasonic sensor and technology for diagnosing and optimizing the process of water freezing to save energy.

## 1. Introduction

The freeze drying process, also known as lyophilization, is a critical and well-established process for the long-term storage of food, drug, biopharmaceutical products, *etc.* [1]. Almost 50% of the marketed biopharmaceuticals are freeze dried, thus indicating that the freeze drying process is the most common formulation strategy [2]. The advantages of this process include better stability, easy handling and storage and good product quality [3].

The traditional freeze drying process is a dehydration process by sublimation of a frozen product, and consists of three steps: freezing, primary drying and secondary drying [4]. In the freezing stage, the liquid is cooled down until ice crystals start to nucleate and grow. Then the crystalline ice forms and is removed by sublimation in the primary drying stage. In this stage, the vacuum chamber pressure is reduced below the vapor pressure of ice, and the shelf temperature is raised to provide the heat for ice sublimation [5]. The remaining 15%–20% water inside the product would be desorbed at elevated temperature and low pressure in the secondary drying stage [6].

The freezing stage is the first and the shortest step of the freeze drying process, but it governs several critical parameters, such as the sublimation and desorption rates [7], and the final quality of the lyophilized product [8]. The initial/end freezing point, freezing rate and degree of supercooling in the freezing stage are important thermodynamic factors for the prediction of thermal and physical properties. Accurate freezing point data can be utilized to determine several properties, such as effective molecular weight, water activity, frozen water, enthalpy below freezing, construction of state diagrams [9], freezing and thawing of frozen foods, *etc.* The freezing point is also important to estimate the freezing time and other structural properties, such as glass transition, end point of freezing, and fraction of unfrozen water in foods. Due to this importance and its wide applications, the estimate of freezing point and modeling these properties are crucial in food processing (freezing and drying) and food stability during storage [10].

The complex interplay of these properties in the freezing stage requires the use of real-time process diagnosis and quality control procedures. Currently, the most widely used freezing process diagnostic tool deploys temperature sensors for online measurement [11]. The temperature sensors, however, need to be in direct contact with the frozen samples in order to provide the temperature information [12], but a sensor embedded in the product may cause removal inconveniences after the freeze drying process. For drug and biopharmaceutical products, the direct contact of a sensor with the products may cause undesired pollution. There are also several off-line measuring methods, such as theoretical thermodynamic calculations, cryo-microscopy, differential scanning calorimetry (DSC), thermo-mechanical analysis (TMA), dynamic mechanical analysis (DMA) and dynamic mechanical thermal analysis (DMTA) [13] for measuring the thermodynamic properties, glass transition, weight, heat flow, dimensional changes, as well as viscoelastic properties of the tested samples or products. All the off-line methods need very costly instruments and operator skill to measure and interpret the data.

The ultrasonic technique is a widely known non-destructive and non-intrusive method for real-time process diagnosis [14]. The basic signatures of ultrasonic signals, such as velocity, attenuation, reflection and transmission coefficients, scatter signals from materials, all have unique relationships with process dynamics [15], material characteristics [16], and product quality [17]. Ultrasonic signals can also reveal the temperature of a material [18,19,20]. Ultrasonic technology has also been adopted for decreasing the sublimation time by controlling the nucleation temperature during the primary drying stage [21], but it has not been utilized for freezing point diagnosis during the freezing process. This study attempts to apply ultrasonic techniques to the real-time diagnosis of freezing processes. In particular, an ultrasonic transducer (UT) is integrated onto the bottom of a freezing bottle. Then, during the process of freezing water at various cooling temperatures, the process is monitored and diagnosed by ultrasonic technology to evaluate the freezing point and cooling/freezing periods to optimize the process and save energy.

## 2. Development of Ultrasound-Assisted Diagnosis System

**Figure 1:**
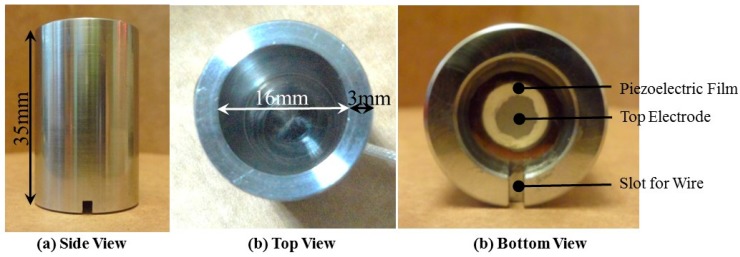
Photographs of the freezing bottle made of stainless steel. (**a**) Side; (**b**) top and (**c**) bottom views.

Since the operating temperature of most ultrasonic couplant is between −18 to 100 °C, and the space available for installing sensors in a freezing machine is limited, the development of UT is one of the key factors to realize the ultrasonic diagnosis of industrial freeze drying processes at freezing temperatures. The procedure for fabricating the UT by a sol-gel spray technique is described in previous publications [22,23,24]. The UT was applicable at temperature range of −100 to 400 °C without an ultrasonic coupler. It could be operated in a medium megahertz (MHz) frequency range with a sufficient frequency bandwidth and had a sufficient piezoelectric strength and signal-to-noise ratio (SNR). Figure 1a presents the utilized freezing bottle made of stainless steel. The height of the freezing bottle is 35 mm. Figure 1b shows the top view of bottle. The inner and outer radii of the bottle are 16 and 22 mm, respectively. The inner height and capacity of the bottle are 30 mm and 6.03 cm^3^, respectively. According to the size of the freezing bottle, the UT sensor was designed with a circular shape with the following dimensions: 8 mm in radius and 103 μm in thickness, as shown in Figure 1c. The top electrode was fabricated with silver paste with the radius of 4 mm. The UT sensor was well aligned on the center of the cavity.

**Figure 2:**
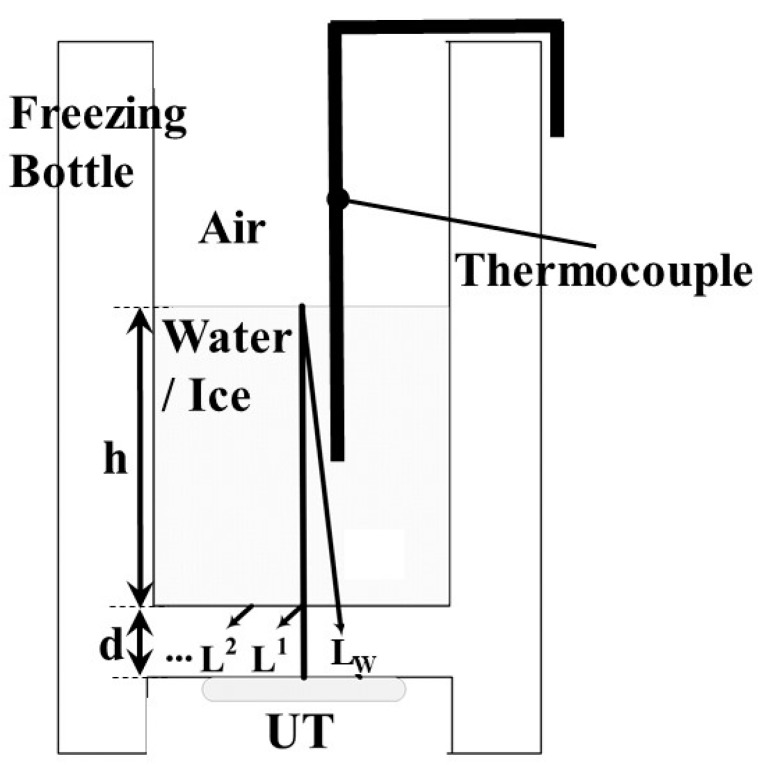
Schematic view of the freezing bottle with UT illustrating the ultrasound transmission paths between the bottle/water or ice. A temperature sensor was set in the middle of the freezing bottle for measuring the water/ice temperature.

A schematic view of the freezing bottle with the UT sensor incorporated in the freeze dryer machine during the freezing process is displayed in Figure 2. As shown in Figure 2, when electric pulses were applied to the piezoelectric film through the top and bottom electrodes, where the freezing bottle itself served as the bottom electrode, ultrasonic waves were excited and transmitted into the freezing bottle. L^n^ (*n* = 1, 2,…) denote the nth round trip longitudinal-wave ultrasonic echoes reflected from the interface of the freezing bottle/water or ice, and L_w_ is the 1st echo propagating in the water and reflected from the water/air interface. The L^1^ and L_w_ echoes will be used to monitor the freezing process and water state. The height of water level and thickness of bottle bottom are denoted as h and d, respectively. A temperature sensor (Type T thermocouple, Omega, Stamford, CT, USA) was set in the middle of the freezing bottle for measuring the water/ice temperature. The temperature would be measured for a comparison with the ultrasonic signals during the freezing process.

**Figure 3:**
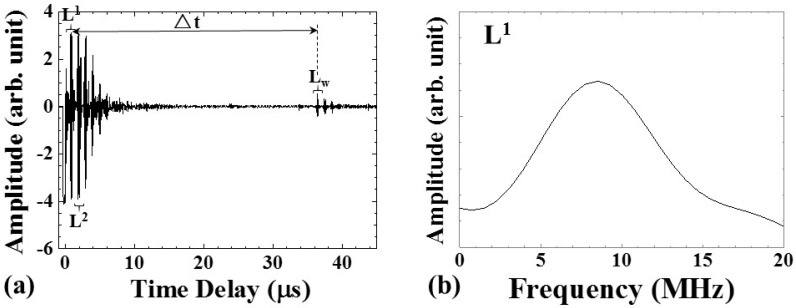
(**a**) Typical ultrasonic signals acquired by UT; (**b**) the frequency spectrum of L^1^ signal. The center frequency of the L^1^ echo was 8.51 MHz, and the 3-dB bandwidth was 8.70 MHz.

Figure 3a shows the typical ultrasonic signals acquired with the UT in Figure 2. As one can see, the L^n^ echoes (*n* = 1, 2, …), reflected at the bottle/water or ice interface, appeared at 0.81 and 1.87 μs, respectively, and remain during the entire process. When the water was not frozen, the L_w_ echo, propagating in the water and reflected at the water/air interface, was observed at 36.39 μs. The time delay difference between the L^1^ and L_w_ echoes was denoted as Δt. Figure 3b shows the frequency spectrum of the L^1^ echo in Figure 3a. The center frequency of the L^1^ echo was 8.51 MHz, and the 3-dB bandwidth was 8.70 MHz. The SNR for the first round trip echo, L^1^, was 37.6 dB. The SNR value could be calculated according to the following equation:

(1) $${\text{SNR}}_{dB}=10log_{10}\left(\frac{P_{L^{1}}}{P_{Noise}}\right)=20log_{10}\left(\frac{A_{L^{1}}}{A_{Noise}}\right)$$

where P and A are the power and amplitude of signals, respectively.

## 3. Experimental Setup

**Figure 4:**
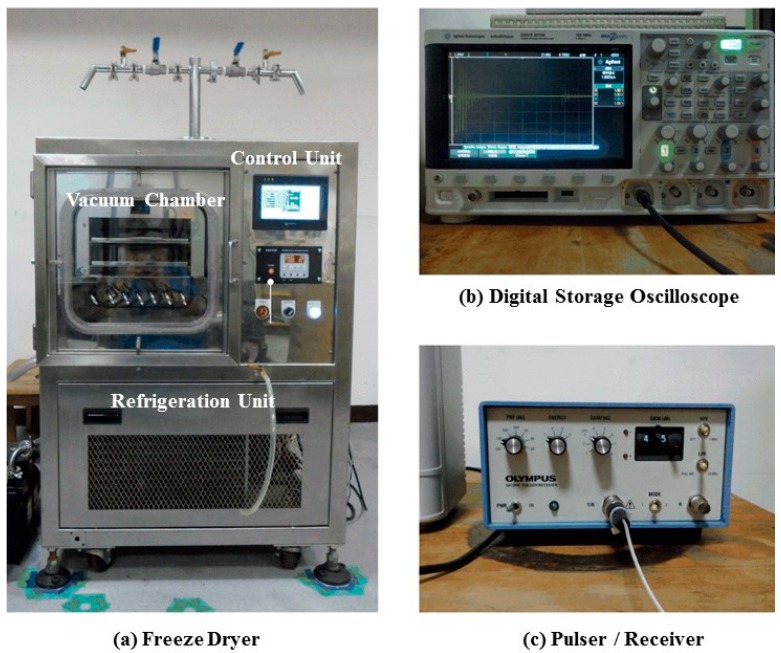
Photographs of (**a**) the freeze dryer machine with vacuum chamber, controller unit, refrigeration unit and vacuum pump; (**b**) the digital oscilloscope and (**c**) the ultrasonic pulser/receiver unit.

In this experiment, a 4 L, air-cooling type shelf freeze dryer machine (TYFD-50005, Tai Yiaeh, New Taipei City, Taiwan), equipped with vacuum chamber, refrigeration and control units, as shown in Figure 4a, was used. The vacuum chamber was for freezing and drying the samples under low temperature (+50~−40 °C) and pressure (760~0.05 torr) conditions.

The steel freezing bottle with UT and thermocouple was set on the shelf of vacuum chamber during the freezing process. The refrigeration unit was for the refrigeration and heat exchange processes of the refrigerant and antifreeze. The control unit comprised a programmable logic controller (PLC) and a human-machine interface for controlling the freeze drying process. The measured temperature data from the thermocouple was recorded by the PLC control unit every second during the freezing process. Figure 4b presents the utilized digital oscilloscope (DSO-X2014A, Agilent Technologies, Santa Clara, CA, USA) for recording the ultrasonic signals during the freezing process. The digital oscilloscope has four channels, 100 MHz bandwidth, a maximum sampling rate of 2 GSa/s and a maximum memory depth of 100 kpts/channel. Figure 4c presents the utilized pulser/receiver (5072PR, Olympus, Tokyo, Japan), which is a broadband, negative spike pulser and broadband receiver. It can be applied in reflection or transmission mode. For the pulser, the pulse voltage under no load is −360 V and the pulse repetition rate is 100~5000 Hz. For the receiver, the broad bandwidth is 1 kHz~35 MHz, with high/low pass filters of 1 kHz~1 MHz and 10~35 MHz, respectively.

The freezing bottle was filled with a certain amount of water at room temperature, and then installed on the shelf of the freeze dryer machine. The freezing bottle with water would be cooled down to the shelf temperature. After the temperature of the freezing bottle with ice reached a stable state, the experiment was stopped. The designed water levels were 5, 15 and 25 mm. The temperature settings of the shelf were −20, −30 and −40 °C. The air pressure of the vacuum chamber was 101.3 kPa (1 atm). The liquid utilized to fill the freezing bottle was water. All the experiments presented in this study were conducted in the ultrasonic pulse-echo mode. The ultrasonic signals were acquired every 5 s in this paper.

## 4. Results and Discussion

### 4.1. Freezing Process Diagnosis by Temperature Measurements and Visual Observations

**Figure 5:**
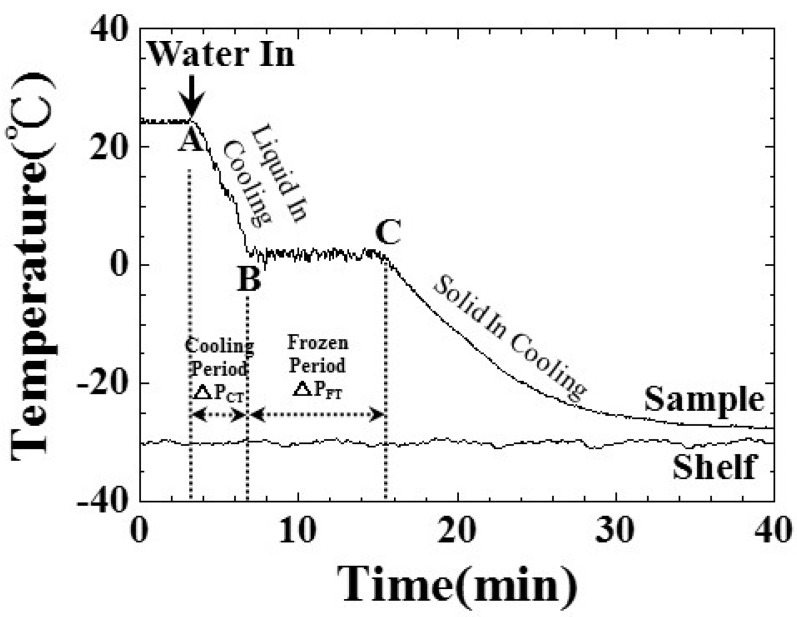
Variations of the shelf and water temperature during the freezing process. The shelf temperature was set as −30 °C, and the water level was 25 mm.

Temperature variation during the freezing process has a close relationship with the rate of freezing, and it would affect the size of the ice crystal nuclei and the quality of the freezing process. In order to investigate the correlation between the water temperature and the water freezing process, the water temperature variation inside the freezing bottle with respect to the process time was determined. The water was filled to a level of 25 mm in the freezing bottle at room temperature and an air pressure of 101.3 kPa. The water and shelf temperatures recorded by the temperature sensors are shown in Figure 5. The shelf temperature was set at −30 °C throughout the entire process. At a process time of 3.2 min, the freezing bottle was put into the freeze dryer machine and the temperature of water started to drop. This point was denoted as point A. From 3.2 to 6.9 min, the water temperature was cooled down from 24.1 to 1.4 °C, with a rate of decrease of 6.14 °C/min. During this period, the sensible heat of water was removed. This period is denoted as the cooling period, ΔP_CT_, measured by the thermocouple. From 6.9 to 15.2 min, the water temperature was kept within the range of 0.7 to 2.9 °C. During this period, the latent heat of water was removed through the bottle/shelf interface, and the water would change phase from liquid to solid (ice). The points at the process times of 6.9 and 15.2 min would be denoted as points B and C [11], respectively, indicating the freezing point and phase change end of water. This period is also denoted as the freezing period, ΔP_FT_, measured by the thermocouple. Then, from 15.2 to 40 min, the water temperature was cooled down again from 1.2 to −27.6 °C for further remove the sensible heat of ice. The freezing process stopped at the process time of 40 min when the ice temperature was −27.6 °C.

**Figure 6:**
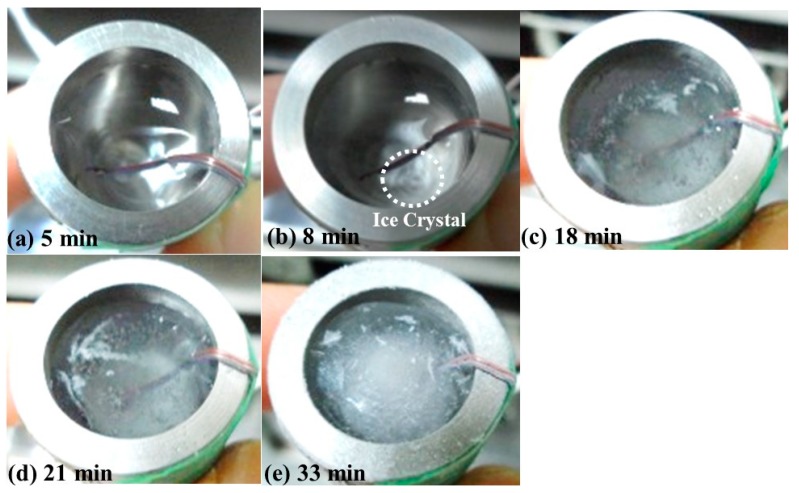
Photographs of the freezing bottle with water/ice during the freezing process in Figure 5 at the process times of (**a**) 5; (**b**) 8; (**c**) 18; (**d**) 21 and (**e**) 33 min, respectively. Water level: 25 mm; room temperature: 25 °C; shelf temperature: −30 °C; air pressure: 101.3 kPa.

Figure 6a–e are photographs of the freezing bottle with water/ice during the freezing process when the process times in Figure 5 were 5, 8, 18, 21 and 33 min, respectively. The freezing process steps presented in Figure 6a–e could be described as follows:

(1) Figure 6a (process time of 5 min, water temperature of 15.7 °C): the freezing bottle was filled with the water and it was installed on the shelf of the freeze dryer machine.
(2) Figure 6b (process time of 8 min, water temperature of 2.9 °C): most of the water was in the liquid state; the water close to the bottom, started to become opaque and some ice crystals were observed beside the thermocouple. This phenomenon may indicate that the water was starting to freeze.
(3) Figure 6c (process time of 18 min, water temperature of −6.3 °C): Most of the water became slush, and the water clarity became worse. This may be due to the appearance of air bubble nucleation in the bottom of the freezing bottle [25,26]. The freezing process seemed to be incomplete.
(4) Figure 6d (process time of 21 min, water temperature of −13.6 °C): Most of the water had become ice, and the air bubbles may extend outwards and upwards from the center bottom, so several air bubbles could be observed close to the surface.
(5) Figure 6e (process time of 33 min, water temperature of −26.6 °C): Most of the air bubbles were pushed to the top of the ice, and the ice surface displayed the cone shape, and the ice became opaque. The freezing process stopped at the process time of 40 min.

### 4.2. Freezing Process Diagnosed by Ultrasonic Signatures

Even though the water freezing process is familiar to most people, however, the diagnosis of this process is typically limited to visual observation and temperature methods. According to the authors’ knowledge, the use of ultrasound technology to monitor the freezing process may be rare.

**Figure 7:**
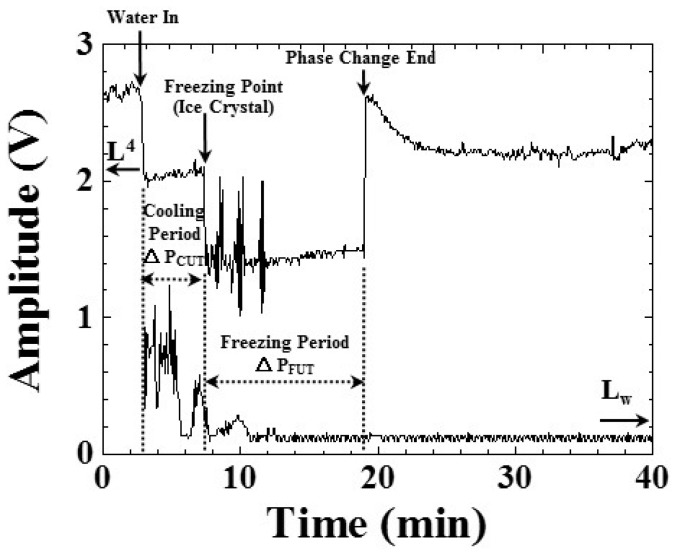
Amplitude variations of ultrasonic L^4^ and L_w_ echoes during the freezing process, indicating the water-in, freezing point (ice crystals) and phase change end during the freezing process. Water level: 25 mm; room temperature: 25 °C; shelf temperature: −30 °C; air pressure: 101.3 kPa.

In order to investigate the correlation between the ultrasonic signals observed and the water freezing process, the amplitude values of the L^4^ and L_w_ echoes in Figure 3a with respect to the process time were obtained. The results are presented in Figure 7. The reason of choosing ultrasonic echo L^4^, instead of L^1^, is that the L^4^ echo is more sensitive to the variation of steel/water interface. The amplitude variations of the ultrasonic L^4^ and L_w_ echoes, corresponding to the freezing process in Figure 6, are described as follows:

(1) Process time of 2.75 min, temperature of water 24.2 °C: the freezing bottle was filled with water to a level of 25 mm at room temperature (25 °C) and an air pressure of 101.3 kPa. The shelf temperature was set as −30 °C. At this moment, the amplitude of the L^4^ echo decreased and the amplitude of the L_w_ echo increased, due to the fact that a part of the ultrasonic energy was transmitted into the water through the steel bottle/water interface.
(2) Process time of 7.51 min, temperature of water 1.7 °C: the amplitudes of the L^4^ and L_w_ echoes decreased, due to the appearance of ice crystals and variation of the water’s acoustic impedance, as shown in Figure 6b. The period from water-in to the amplitude decrease of L^4^ echo (*i.e.*, freezing point, appearance of ice crystals) could be denoted as the cooling period, ΔP_CUT_, indicating the period for cooling the water in the freezing bottle. At the process times of 8.50, 10.16 and 11.59 min, the amplitude of the L^4^ echo increased. These phenomena may be due to the non-uniform freezing of the water.
(3) Process time of 19.08 min, temperature of water −10.9 °C: the amplitude of the L^4^ echo increased suddenly. This may be due to the freezing of water and the appearance and extension of air bubbles, as shown in Figure 6d,e. The extension of air bubbles would also change the acoustic impedance of ice locally. The period from the freezing point to the increase of L^4^ echo amplitude could be denoted as the freezing period, ΔP_FUT_, indicating the period for freezing the liquid in the freezing bottle.
(4) After process time of 19.08 min: the amplitude of the L^4^ echo decreased slightly and reached a stable state at the process time of 24.5 min. This may be due to the fact that most of the air bubbles were pushed to the top of the ice. Therefore, the ice close to the bottom became more solid, and the acoustic impedance of ice changed again. The experiment was stopped at the process time of 40 min.

**Figure 8:**
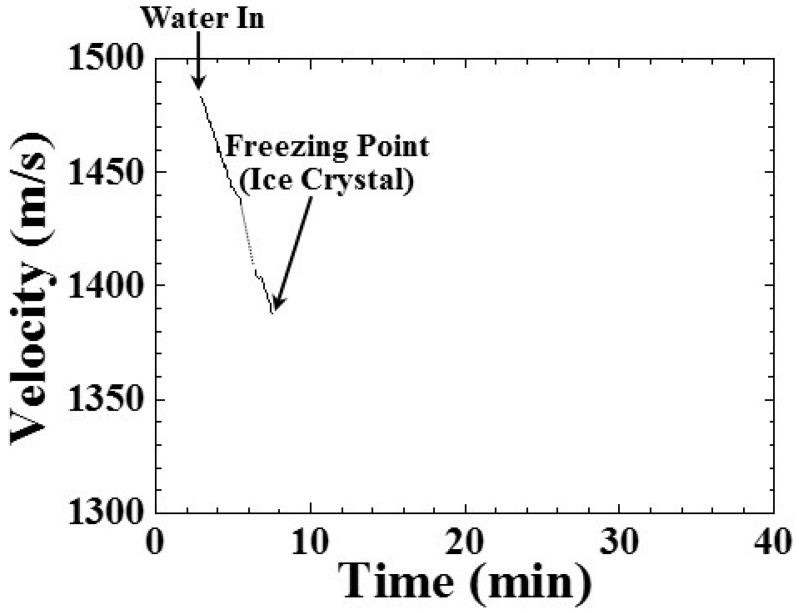
Variation of the ultrasonic velocity in the water of the freezing bottle, indicating the water-in, cooling, and freezing point (appearance of ice crystals) during the freezing process. Water level: 25 mm; room temperature: 25 °C; shelf temperature: −30 °C; air pressure: 101.3 kPa.

During the freezing process, the speed of freezing of water would affect the size of ice crystal nuclei and the frozen water quality. Ultrasonic velocity may be one of the candidates to indicate the freezing speed of water, because of its close relationship with the temperature. The ultrasonic velocity in the water could be calculated according to the following equation:

(2) $${\text{v}}_{\text{w}}\;=\;2\text{h}/\text{Δt}$$

where h is the height of the water level in Figure 2 and Δt is the time delay between the ultrasonic echoes L^1^ and L_w_ in Figure 3a. The result is shown in Figure 8.

In Figure 8, the ultrasonic velocity appeared at a process time of 2.91 min, when the freezing bottle was filled with at a room temperature of 25 °C and an air pressure of 101.3 kPa. The shelf temperature was set as −30 °C. At this moment, the ultrasonic velocity in the water was 1483.7 m/s. From a process time of 2.91 to 7.50 min, the ultrasonic velocity decreased from 1483.7 to 1387.0 m/s, indicating that the temperature of water decreased during the freezing process. The decreasing tendency could be expressed in the following equation:

(3) $${\text{v}}_{\text{w}}\;=\;1544.88-20.76\times (\text{t}-2.91)$$

where v_w_ (m/s) is the ultrasonic velocity in water, and t is the process time. From a process time of 5.58 to 6.41 min, the ultrasonic velocity disappeared due to the missing L_w_ echo. At a process time of 7.50 min, the ultrasonic velocity disappeared due to the amplitude of the L_w_ echo decreasing to the noise level. The ultrasonic velocity decreased with the water temperature. The rate of decrease of the ultrasonic velocity in water was 20.76 (m/s)/min and 4.3 (m/s)/°C. Therefore, the cooling speed of water could be diagnosed through the amplitude and velocity variation of the ultrasonic signals.

### 4.3. Effect on Freezing of Various Water Amounts

**Figure 9:**
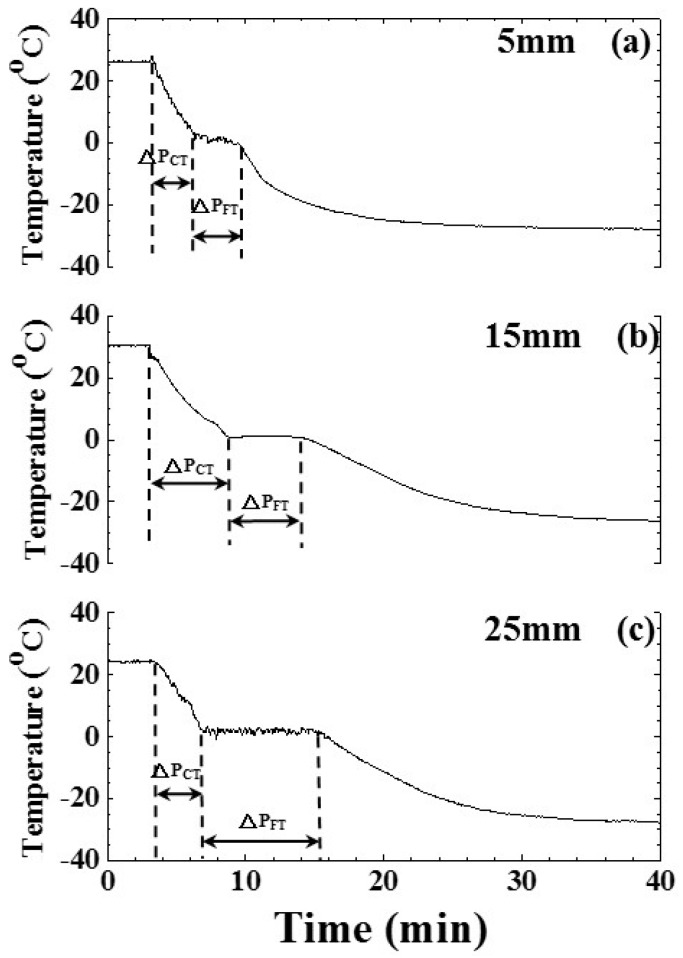
Effect of water level on temperature during the freezing process. Water level: (**a**) 5; (**b**) 15; and (**c**) 25 mm. Shelf temperature: −30 °C; air pressure: 101.3 kPa.

**Figure 10:**
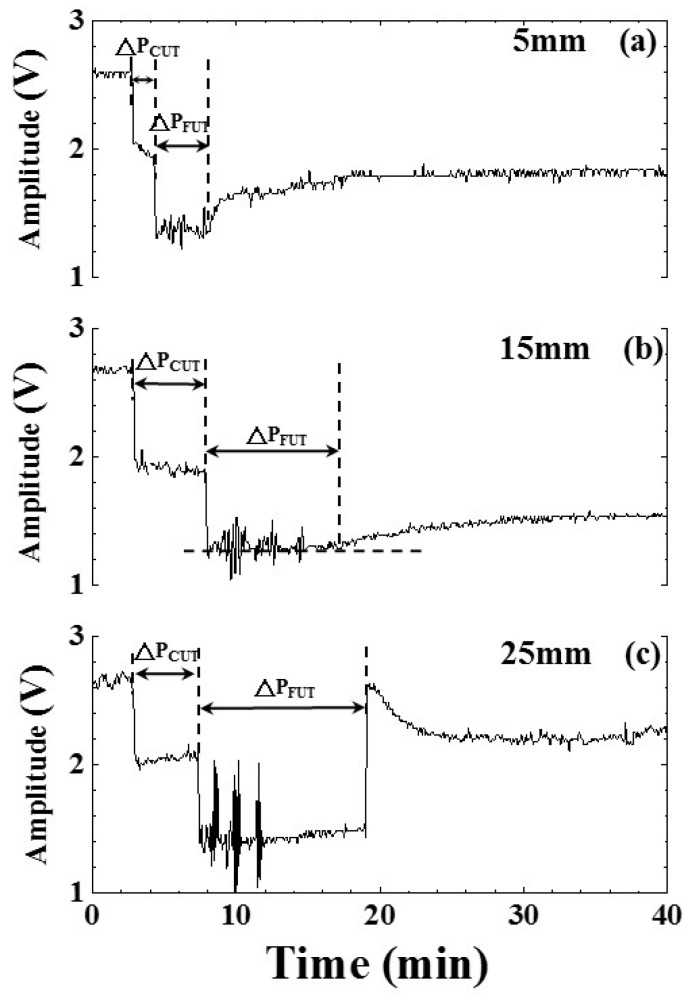
Effect of water level on amplitude variation of ultrasonic L^4^ echo during the freezing process. Water level: (**a**) 5; (**b**) 15; and (**c**) 25 mm. Shelf temperature: −30 °C; air pressure: 101.3 kPa.

A linear relationship between the cooling/freezing period, indicated by the ultrasonic signature, and the water level would be a fundamental requirement to utilize this technology. To study this relationship, the shelf temperature and air pressure were set at −30 °C and 101.3 kPa, respectively, to freeze various water levels of 5, 15, 25 mm. The experimental results of water temperature and amplitude of the ultrasonic L^4^ echo related to water levels of 5, 15, 25 mm ae shown in Figure 9 and Figure 10, respectively. Figure 9a–c are the temperature variation of water in the freezing bottle for water levels of 5, 15, 25 mm, respectively. The timings of the freezing point and phase change end indicated by the temperature occurred at the process times of 6.30, 8.73, 6.98 and 9.48, 14.65, 15.98 min for the water levels of 5, 15 and 25 mm, respectively. When the water level was lower, the timing of the phase change end indicated by temperature was earlier. The cooling/freezing periods of various experimental conditions were also indicated.

The corresponding temperature and timing of water-in, freezing point and phase change end indicated by temperature are listed in Table 1. It seemed that there was a linear relationship between the timings of freezing point/phase change end and the water level.

**Table 1:** Timings and temperatures of water in, freezing point and phase change end indicated by temperature for water levels of 5, 15, 25 mm. Shelf temperature: −30 °C; air pressure: 101.3 kPa.

Water Level (mm)	Status	Water in	Freezing Point	Phase Change End
5	Time (min)	3.28	6.3	9.48
	Temperature (°C)	27.3	0.9	−0.2
15	Time (min)	3.05	8.73	14.65
	Temperature (°C)	30.6	1.0	−0.2
25	Time (min)	3.21	6.98	15.98
	Temperature (°C)	24.5	1.3	−0.4

Figure 10a–c are the amplitude variation of the ultrasonic L^4^ echo during the freezing process for water levels of 5, 15, 25 mm, respectively. The freezing point and phase change end indicated by the ultrasonic L^4^ echo occurred at the process times of 4.25, 7.83, 7.33 and 8.25, 17.42, 19.08 min for the water levels of 5, 15 and 25 mm, respectively. When the water level was lower, the timing of the phase change end indicated by the ultrasonic L^4^ echo was earlier. The cooling/freezing periods of various experimental conditions were also indicated. The corresponding timings of water-in, freezing point and phase change end indicated by ultrasonic L^4^ echo are given in Table 2. It seemed that there was a linear relationship between the timings of freezing point/phase change end and the water level.

**Table 2:** Timings of water in, freezing point and phase change end indicated by ultrasonic L^4^ echo for water levels of 5, 15, and 25 mm. Shelf temperature: −30 °C; air pressure: 101.3 kPa.

Water Level (mm)	Timing (min)		
	Water in	Freezing Point	Phase Change End
5	2.75	4.25	8.25
15	2.83	7.83	17.42
25	2.83	7.33	19.08

**Figure 11:**
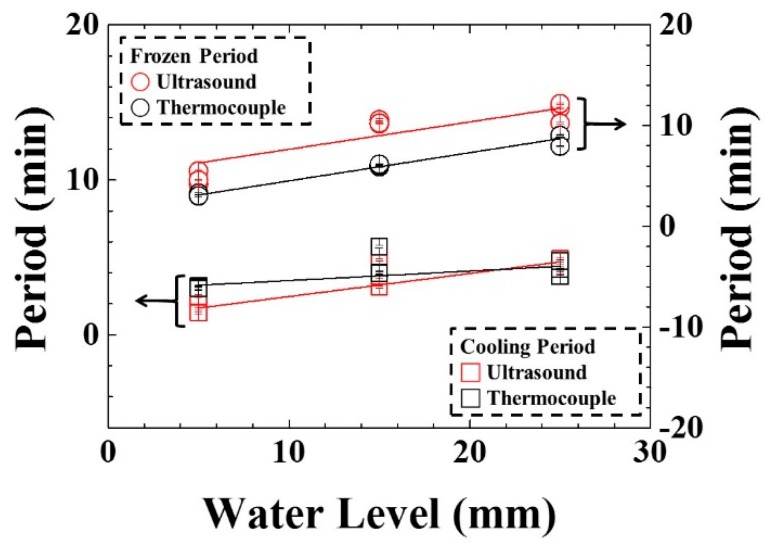
Variation of cooling/freezing period with various water levels. Water level: 5, 15, 25 mm; shelf temperature: −30 °C; air pressure: 101.3 kPa.

In order to view clearly the mentioned linear relationship, the cooling/freezing periods were compared with the water level. The results are shown in Figure 11. The cooling/freezing periods were marked by square and circle symbols, respectively. Those indicated by temperature and ultrasound were marked by black and red color, respectively. The estimated errors of cooling/freezing periods for the experimental conditions were less than 3%. In the water level range from 5 to 25 mm, the average cooling/freezing periods indicated by ultrasonic L^4^ echo increased from 1.73 to 4.73 min and from 6.28 to 11.68 min linearly, respectively. The straight line for these symbols was obtained by a least squares fitting method, which is explained in the following section. The slopes of the fitting lines were 0.15 and 0.27 min/mm for the cooling/freezing periods, respectively. The cooling/freezing periods can be expressed as:

(4) $$\text{Δ}{\text{P}}_{CUT}=0.98+0.15\times \text{h}$$

(5) $$\text{ Δ}{\text{P}}_{FUT}=4.93+0.27\times \text{h}$$

where ΔP_CUT_ and ΔP_FUT_ are the cooling and freezing periods in Figure 10, respectively, and h is the water level in Figure 2. In the water level range from 5 to 25 mm, the average cooling/freezing periods indicated by temperature increased from 3.19 to 4.39 min and from 3.12 to 8.72 min, respectively. The slopes of the fitting lines were 0.06 and 0.28 min/mm for the cooling/freezing periods, respectively. The cooling/frozen periods can be expressed as:

(6) $$\text{Δ}{\text{P}}_{CT}=2.89+0.06\times \text{h }$$

(7) $$\text{Δ}{\text{P}}_{FT}=1.72+0.28\times \text{h}$$

where ΔP_CT_ and ΔP_FT_ are the cooling and freezing periods in Figure 9, respectively. This indicated that a higher level of water would result in a longer cooling/freezing periods. The different slopes of the cooling and freezing periods were due to the various specific heat capacity caused by the latent and sensible heat of water. Therefore, the ultrasonic technique can clearly indicate the cooling/freezing completion at each water level to shorten the freezing process and save energy.

### 4.4. Freezing Effect of Various Freezing Speeds

**Figure 12:**
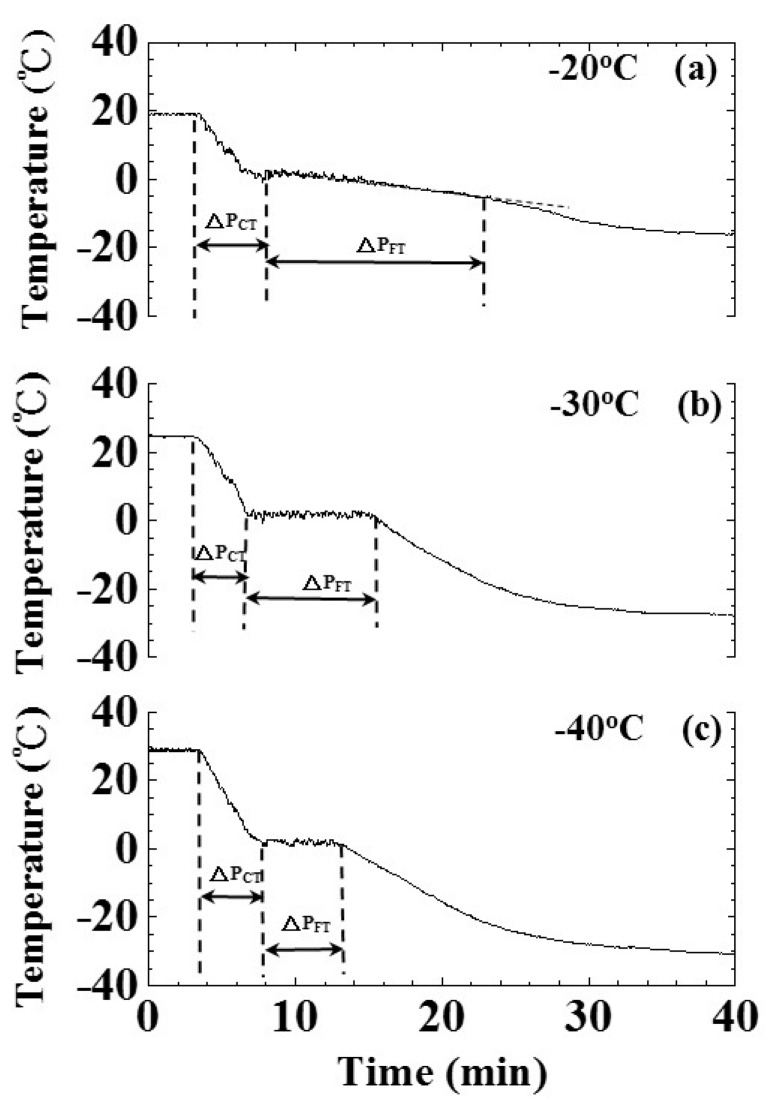
Effect of freezing speed on temperature during the freezing process. Shelf temperatures: (**a**) −20; (**b**) −30 and (**c**) −40°C. Water level: 25 mm; air pressure: 101.3 kPa.

**Figure 13:**
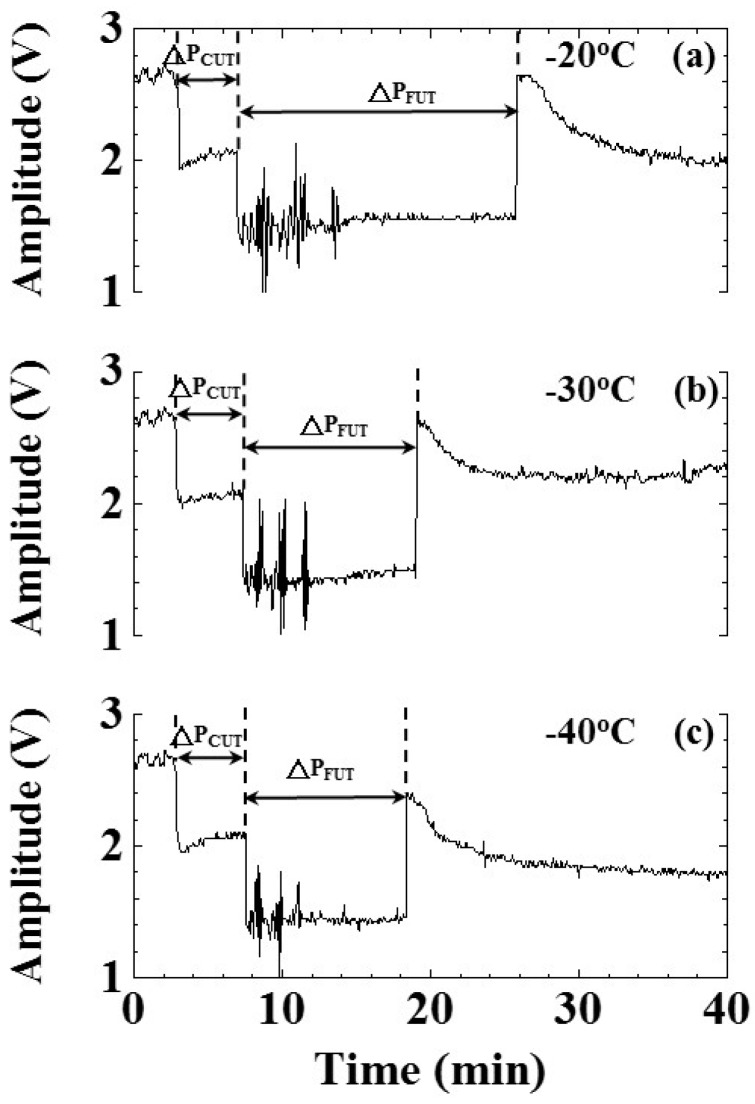
Effect of freezing speed on amplitude variation of ultrasonic L^4^ echo during the freezing process. Shelf temperatures: (**a**) −20; (**b**) −30 and (**c**) −40 °C. Water level: 25 mm; air pressure: 101.3 kPa.

The freezing speed would affect the size of ice crystal nuclei and the frozen ice quality. We were interested in evaluating the effect of freezing speed on cooling/freezing periods indicated by ultrasonic signatures. To study this effect, the water level and air pressure were set at 25 mm and 101.3 kPa, respectively, under the shelf temperatures of −20, −30 and −40 °C. The lower shelf temperature represents the larger temperature difference and the faster cooling/freezing speed. The freezing speed of the freeze dryer machine was controlled by the programmable logical controller (PLC) under the setting of the PID controlling rule. The experimental results of water temperature and amplitude of ultrasonic L^4^ echo related to shelf temperatures of −20, −30 and −40 °C are shown in Figure 12 and Figure 13, respectively. Figure 12a–c are the temperature variation of water in the freezing bottle for shelf temperatures of −20, −30 and −40 °C, respectively. The freezing point and phase change end indicated by temperature occurred at the process times of 7.80, 6.98, 7.83 and 23.43, 15.98, 13.22 min for the shelf temperatures of −20, −30 and −40 °C, respectively.

When the shelf temperature was low, the timing of the phase change end indicated by temperature was earlier. The cooling/freezing periods of various experimental conditions were also indicated. The corresponding temperature and timing of water-in, freezing point and phase change end indicated by temperature are illustrated in Table 3.

**Table 3:** Timings and temperatures of water in, freezing point and phase change end indicated by temperature for shelf temperatures of −20, −30 and −40 °C. Water level: 25 mm; air pressure: 101.3 kPa.

Shelf Temperature (°C)	Status	Water in	Freezing Point	Phase Change End
−20	Time (min)	3.50	7.80	23.43
	Temperature (°C)	19.0	−0.9	12.7
−30	Time (min)	3.21	6.98	15.98
	Temperature (°C)	24.5	1.3	−0.4
−40	Time (min)	3.40	7.83	13.22
	Temperature (°C)	28.8	0.8	−0.3

Figure 13a–c are the amplitude variation of the ultrasonic L^4^ echo during the freezing process for shelf temperatures of −20, −30 and −40 °C, respectively. The timings of freezing point and phase change end indicated by the ultrasonic L^4^ echo occurred at the process times of 6.91, 7.33, 7.50 and 25.83, 19.08, 18.41 min for the shelf temperatures of −20, −30 and −40 °C, respectively. When the shelf temperature was low, the timing of the phase change end indicated by the ultrasonic L^4^ echo was earlier. The cooling/freezing periods of various experimental conditions were also indicated. The corresponding timing of water-in, freezing point and phase change end indicated by the ultrasonic L^4^ echo are illustrated in Table 4.

**Table 4:** Timings of water in, freezing point and phase change end indicated by ultrasonic L^4^ echo for shelf temperatures of −20, −30 and −40 °C. Water level: 25 mm; air pressure: 101.3 kPa.

Shelf Temperature (°C)	Timing (min)		
	Water in	Freezing Point	Phase Change End
−20	3.00	6.91	25.83
−30	2.83	7.33	19.08
−40	2.75	7.50	18.41

**Figure 14:**
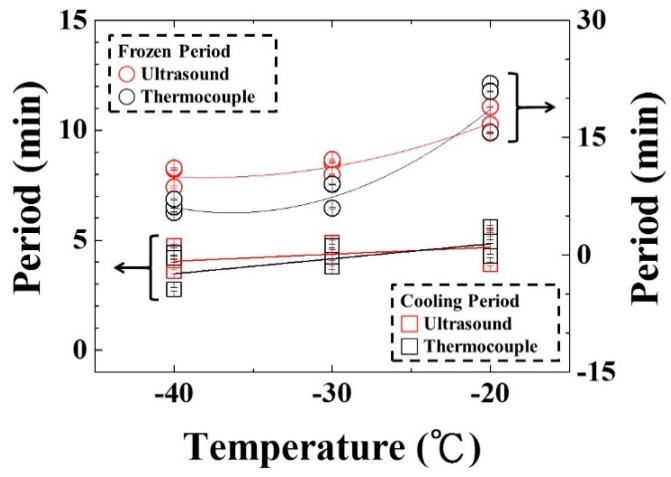
Variation of cooling/freezing period with various shelf temperatures. Shelf temperature: −20, −30 and −40 °C; water level: 25 mm; air pressure: 101.3 kPa.

In order to clearly view their relationships, the cooling/freezing periods were compared with the shelf temperature. The results were shown in Figure 14. The cooling/freezing periods were marked by square and circle symbols, respectively. Those indicated by temperature and ultrasound were marked by black and red color, respectively. The estimated errors of cooling/freezing periods for the experimental conditions were less than 3%, respectively. In the shelf temperature range from −40 to −20 °C, the cooling period indicated by temperature and ultrasonic L^4^ echo increased from 4.8 to 3.4 min and from 4.72 to 4.12 min linearly, respectively. The slopes of the fitting lines were 0.07 and 0.03 min/°C for temperature and ultrasonic L^4^ echo, respectively. The cooling period can be expressed as:

(8) $$\text{Δ}{\text{P}}_{CT}=6.20+0.07\times \text{T}$$

(9) $$\text{Δ}{\text{P}}_{CUT}=5.32+0.03\times T\;$$

where ΔP_CT_ and ΔP_CUT_ are the cooling period in Figure 12 and Figure 13, respectively, and T is the shelf temperature. In the shelf temperature range from −40 to −20 °C, the freezing period indicated by temperature and ultrasonic L^4^ echo increased monotonically from 5.39 to 15.63 min and from 10.91 to 18.92 min, respectively. The freezing period seemed reaching a saturated value when the shelf temperature was less than −30 °C. This indicated that the higher shelf temperature would result in the longer cooling/freezing periods. Therefore, the ultrasonic technique can clearly indicate the cooling/freezing completion at each shelf temperature for reducing the freezing process duration and saving energy.

## 5. Conclusions

The freeze drying process is a critical and well-established process for long-term storage of food, drugs, biopharmaceutical products, *etc.* The freezing stage is the first and the shortest step of the freeze drying process, but governs several critical parameters and the final quality of the lyophilized product. The ultrasonic transducer technology is one of the most effective and efficient tools for real-time, non-intrusive and non-destructive monitoring. In this study, an integrated UT was utilized in a stainless bottle for real-time diagnosis of the freezing process of water. The sensor was directly deposited onto the stainless bottle by using a sol-gel spray technique. It could operate at a temperature range from −100 to 400 °C and uses an ultrasonic pulse-echo technique. The progression of the freezing process, including water-in, freezing point and water phase change end, was clearly observed using ultrasound. The ultrasonic signals could indicate the three stages of the freezing process and evaluate the cooling and freezing periods under various processing conditions. The temperature was also adopted for evaluating the cooling and freezing periods. The cooling/freezing periods, indicated by ultrasonic signals and temperature, increased with water volume with the ratio of 0.15 and 0.27 min/mm and 0.06 and 0.28 min/mm, respectively. The cooling period, indicated by temperature and ultrasonic L^4^ echo, decreased with shelf temperature with the ratio of 0.07 and 0.03 min/°C. The freezing period seemed to reach a saturation value when the shelf temperature was less than −30 °C. This study demonstrates the effectiveness of the ultrasonic sensors and technology for diagnosing and optimizing the process of water freezing to save energy.

## References

[1] Tang X., Pikal M. (2004). Design of freeze-drying processes for pharmaceuticals: Practical advice. Pharm. Res..

[2] Constantino H.R., Constantino H.R. (2004). Excipients of use in lyophilized pharmaceutical peptide, protein, and other bioproducts. Lyophilization of Biopharmaceuticals.

[3] Kasper J.C., Friess W. (2011). The freezing step in lyophilization: Physico-Chemical fundamentals, freezing methods and consequences on process performance and quality attributes of biopharmaceuticals. Eur. J. Pharma Biopharma.

[4] Ratii C. (2001). Hot air and freeze-drying of high-value foods: A review. J. Food Eng..

[5] Liu J., Viverette T., Virgin M., Anderson M., Dalal P. (2005). A study of the impact of freezing on the lyophilization of a concentrated formulation with a high fill depth. Pharm. Dev. Technol..

[6] Pikal M.J., Rambhatla S., Ramot R. (2002). The impact of the freezing stage in lyophilization: Effects of the ice nucleation temperature on process design and product quality. Am. Pharm. Rev..

[7] Searles J.A., Carpenter J.F., Randolph T.W. (2001). The ice nucleation temperature determines the primary drying rate of lyophilization for samples frozen on a temperature-controlled shelf. J. Pharm. Sci..

[8] Sarciaux J., Mansour S., Hageman M.J., Nail S.L. (1999). Effects of Buffer Composition and Processing Conditions on Aggregation of Bovine IgG During Freeze-Drying. J. Pharm. Sci..

[9] Rahman M.S. (1995). Food Properties Handgbook.

[10] Rahman M.S. (1999). Glass transition and other structural changes in foods. Handbook of Food Preservation.

[11] Rahman M.S., Guizani N., Al-Khaseibi M., Al-Hinai S.A., Al-Maskri S.S., Al-Hamhani K. (2002). Analysis of cooling curve to determine the end point of freezing. Food Hydrocoll..

[12] Coates P., Haynes A., Speight R. (1994). In-line characterization of polymer deformation in melt and solid phase processing. Polymer.

[13] Roos Y.H., Karel M., Kokini J.L. (1996). Glass transitions in low moisture and frozen foods: Effects on shelf life and quality. Food Technol..

[14] Brown E.C., Collins T.L.D., Dawson A.J., Olley P., Coates P.D. (1999). Ultrasound: A virtual instrument approach for monitoring of polymer melt variables. J. Reinf. Plast. Comp..

[15] Cheng C.C., Ono Y., Jen C.K. (2007). Real-time diagnosis of co-injection molding using ultrasound. Polym. Eng. Sci..

[16] Ono Y., Cheng C.C., Kobayashi M., Jen C.K. (2005). Real-Time Monitoring of Injection Moulding for Microfluidic Devices using Ultrasound. Polym. Eng. Sci..

[17] Wen S.S.L., Jen C.K., Nguyen K.T. (1999). Advances in on-line ultrasonic monitoring of the injection molding process using ultrasonic techniques. Int. Polym. Process..

[18] Takahashi M., Ihara I. (2008). Ultrasonic monitoring of internal temperature distribution in a heated material. Jpn. J. Appl. Phys..

[19] Wadley H.N.G., Norton S.J., Mauer F., Droney B., Ash E.A., Sayers C.M. (1986). Ultrasonic measurement of internal temperature distribution. Phil. Trans. R. Soc. Lond. A.

[20] Kažys R., Voleišis A., Voleišienė B. (2008). High temperature ultrasonic transducers: Review. Ultragarsas (Ultrasound).

[21] Passot S., Tréléa I.C., Marin M., Galan M., Morris G.J., Fonseca F. (2009). Effect of controlled ice nucleation on primary drying stage and protein recovery in vials cooled in a modified freeze-dryer. J. Biomech. Eng..

[22] Kobayashi M., Jen C.K. (2004). Piezoelectric thick bismuth titanate/PZT composite film transducers for smart NDE of metals. Smart Mater. Struct..

[23] Cheng C.C., Ho T.-T. (2010). Dielectric characteristics and orientation of piezoelectric (PbZr_x_Ti_1-x_O_3_, PZT) films fabricated by sol-gel spray and spin techniques. J. Supercond. Novel Magn..

[24] Inoue T., Kobayashi M. (2014). PbTiO_3_/Pb(Zr,Ti)O_3_ sol–gel composite for ultrasonic transducer applications. Jpn. J. Appl. Phys..

[25] Maeno N. (1967). Air bubble formation in ice crystals. Phys. Snow Ice Proceed..

[26] Tao T., Peng X.F., Lee D.J. (2004). Force of a gas bubble on a foreign particle in front of a freezing interface. J. Colloid Interface Sci..

